# Unexpected journey: a colon adenocarcinoma metastatic to the heart

**DOI:** 10.1093/jscr/rjaf379

**Published:** 2025-06-17

**Authors:** Elias Edward Lahham, Abdulla Amro, Hussain Mashaqi, Mahmoud Ramahi, Mohand Abulihya, Hanna Qahoush

**Affiliations:** Department of Radiation Oncology, Augusta Victoria Hospital, Martin Buber Jerusalem 9119101, East Jerusalem, Palestinian Authority, Palestine; Department of Oncology, Augusta Victoria Hospital, Martin Buber Jerusalem 9119101, East Jerusalem, Palestinian Authority, Palestine; Department of Oncology, Augusta Victoria Hospital, Martin Buber Jerusalem 9119101, East Jerusalem, Palestinian Authority, Palestine; Department of Radiology, Augusta Victoria Hospital, Martin Buber Jerusalem 9119101, East Jerusalem, Palestinian Authority, Palestine; Department of Pathology, Al-Estishari Hospital, Ramallah, PO Box 502, West Bank 00970, State of Palestine; Department of Oncology, Augusta Victoria Hospital, Martin Buber Jerusalem 9119101, East Jerusalem, Palestinian Authority, Palestine

**Keywords:** cardiac metastasis, colon adenocarcinoma, intracardiac tumor, metastatic colorectal cancer

## Abstract

Cardiac metastasis from colon adenocarcinoma is a notably rare occurrence but one that warrants detailed discussion due to its complex clinical presentation and significant implications for patient management. The literature on this topic is sparse, with only 31 reported cases, underscoring its rarity and the challenges in developing standardized treatment protocols. This study presents a 53-year-old female patient who was suffering from metastatic adenocarcinoma of the cecum that included the right ventricle. The patient presented with shortness of breath on minimal exertion. The echocardiogram showed a right ventricle filling defect, histopathology confirms the diagnosis of metastatic adenocarcinoma from gastrointestinal origin. This report highlights the importance of recognizing intracardiac metastasis in colon cancer, providing valuable insights for enhanced understanding and potential guidance for future clinical decisions. Cardiac metastasis carries a poor prognosis, emphasizing the need for tailored management strategies.

## Introduction

Colon cancer is the third most prevalent cancer worldwide [1]. Metastases from colon carcinoma typically occur in the lymph nodes, liver, and lungs, while cardiac metastases are exceedingly rare, with autopsy studies showing an incidence of 1.4%–7.2% [2] Although clinical recognition of cardiac metastasis from colorectal cancer is uncommon and remains a diagnostic and therapeutic challenge, this neoplasm should be considered in the differential diagnosis of intracardiac masses [1, 3]. Consequently, describing and evaluating cases of cardiac metastasis related to colon carcinoma is essential for understanding its impact on patient outcomes and developing suitable diagnostic and treatment strategies. In this article, we present a case of colon cancer with cardiac metastasis manifesting as progressive exertional dyspnea.

## Case presentation

A 53-year-old female, a non-smoker with an unremarkable past medical history, presented with a several-month history of abdominal pain and constipation, accompanied by progressive anorexia and weight loss. A rectal examination revealed fecal staining without palpable nodules. Initial laboratory tests showed hemoglobin at 9.5 g/dl, white blood cell count at 9500 cells/mm^3^, and platelets at 440 000 cells/mm^3^. A colonoscopy identified an encircling mass in the cecum, with a biopsy confirming moderately differentiated adenocarcinoma. Elevated serum markers included carcinoembryonic antigen at 51 ng/ml and CA 19–9 at 134 ng/ml.

**Figure 1:**
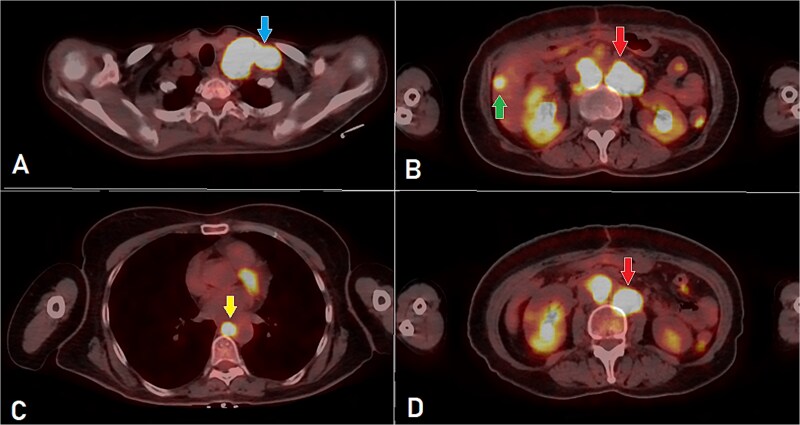
PET-CT scan (A) showing matted intensely hyper-metabolic left supraclavicular lymph nodes (arrow); (B) demonstrating intensely hyper-metabolic retroperitoneal lymph nodes (inferiorly pointing arrow) and a hyper-metabolic solitary hepatic lesion (superiorly pointing arrow); (C) demonstrating an intensely hyper metabolic mediastinal (para- oesophageal – station VIII) lymph node (arrow); and (D) revealing a few intensely hyper-metabolic retroperitoneal lymph nodes (arrow).

**Figure 2:**
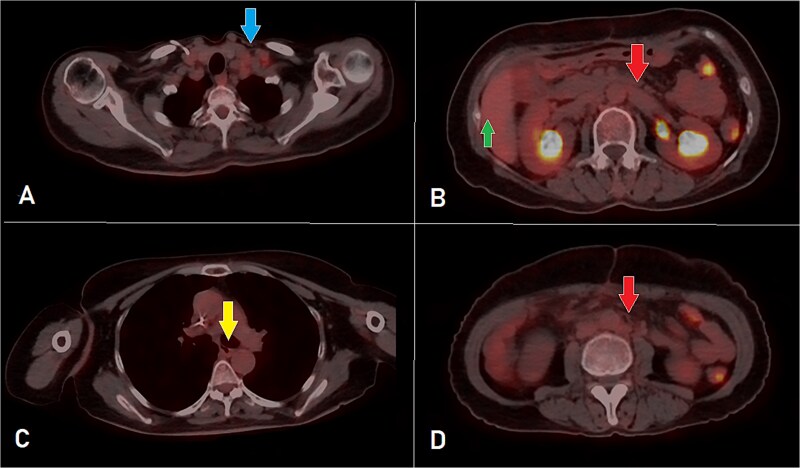
PET-CT scan at the corresponding cuts to Fig. 1 showing great metabolic and morphological regression regarding the previously seen (A) supraclavicular lymph nodes (arrow); (B) retroperitoneal lymph nodes (inferiorly pointing arrow) and hepatic lesion (superiorly pointing arrow); (C) mediastinal (para-oesophageal – station VIII) lymph node (arrow); and (D) retroperitoneal lymph nodes (arrow).

A computed tomography (CT) scan was performed, and the patient underwent a right hemicolectomy using a laparoscopic-assisted approach, with en bloc resection of the tumor and lymphadenectomy. The procedure was uneventful, with no intraoperative complications, and the patient had an unremarkable immediate postoperative recovery. The staging was determined as T4bN2bMx. Further imaging revealed enlarged mediastinal, supraclavicular, and retroperitoneal lymph nodes, with a solitary liver lesion (Fig. 1). A biopsy from the supraclavicular node indicated moderately differentiated adenocarcinoma of gastrointestinal origin. Molecular profiling showed wild-type RAS, intact MSI, mutant BRAF, and HER2 negative. The patient was started on capecitabine-oxaliplatin (CapeOX) with bevacizumab and completed 11 cycles with a marked clinical and radiological response (Fig. 2).

**Figure 3:**
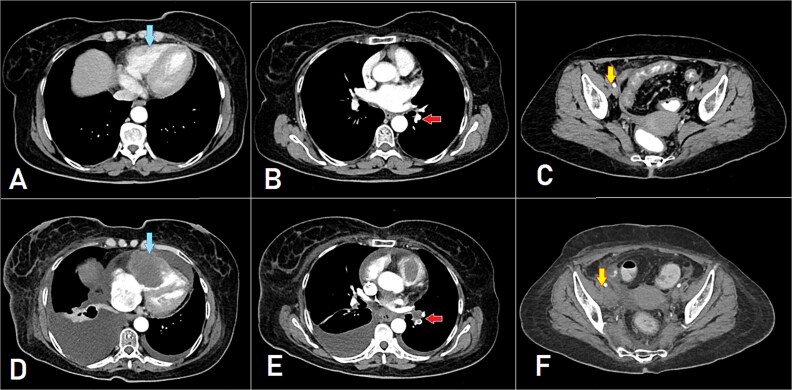
A, B, and C displaying the baseline images. (D) A mass lesion originating from the right ventricle (arrow). (E) Filling defect in the segmental branches of the pulmonary artery representing pulmonary embolism (arrow). (F) A newly seen right para-iliac lymph node (arrow).

After 8 months of CapeOX-bevacizumab treatment, she was switched to DeGramont-bevacizumab due to neuropathy, receiving seven cycles. The patient later elected to discontinue treatment and was lost to follow-up. Seven months later, she presented with dyspnea on minimal exertion. Laboratory tests were within normal limits; however, a CT scan disclosed a new soft tissue density in the right ventricle, measuring up to 6 cm, suggestive of intracardiac involvement. Imaging findings were consistent with a differential of thrombus versus metastatic lesion. Additionally, filling defects of contrast opacification were observed in the right upper and left lower segmental and subsegmental pulmonary artery branches, indicative of concurrent pulmonary embolisms. A new right external iliac lymph node enlargement was also identified. The overall clinical and radiological assessment was consistent with disease progression (Fig. 3).

**Figure 4:**
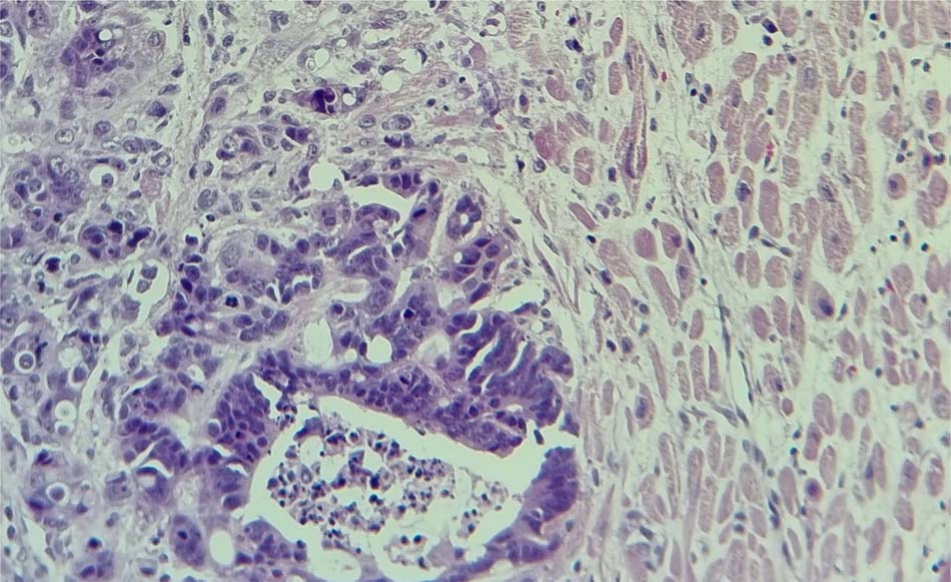
The metastatic tumor cells form glandular structures and infiltrate the myocardial tissue in a disorganized manner.

**Figure 5:**
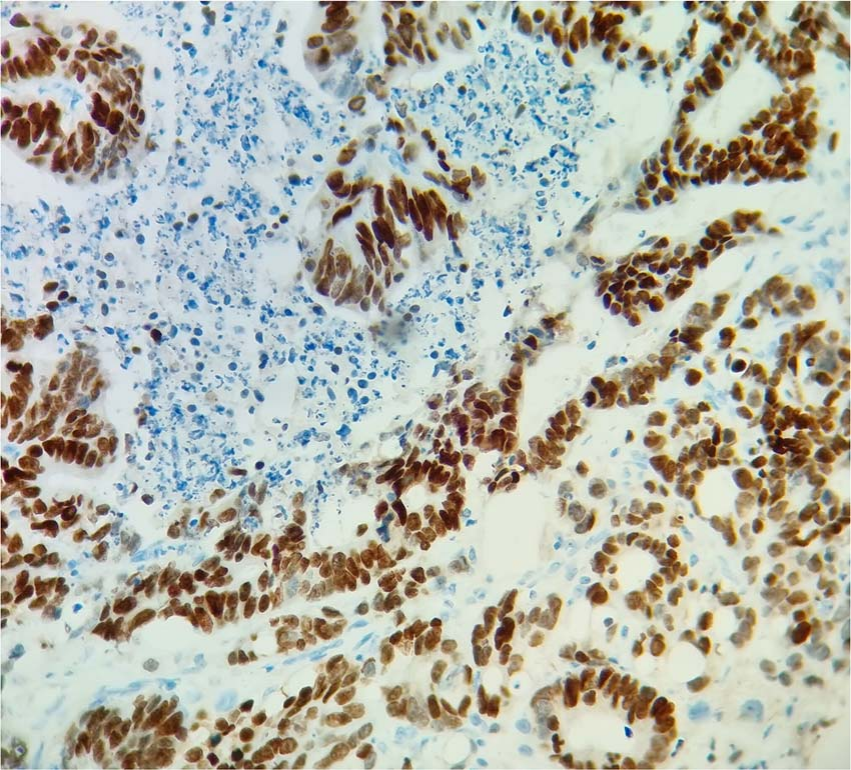
SATB2 immunostain supports the colonic origin of the tumor.

Subsequent echocardiography showed an ejection fraction of 70%, normal left ventricle dimensions, grade II left ventricular diastolic dysfunction, and a mass attached to the right ventricular wall. Mild pulmonary hypertension was noted. The patient underwent an open-heart procedure via median sternotomy under cardiopulmonary bypass. The tumor was adherent to the right ventricular wall, necessitating meticulous dissection to preserve myocardial integrity. The mass was excised completely, and histopathological examination confirmed metastatic adenocarcinoma consistent with a colon primary (Figs 4 and 5). Postoperatively, the patient initially showed stable cardiac function with preserved ejection fraction.

Two months postoperatively, she commenced a new chemotherapy regimen with FOLFIRI-cetuximab. Following the first chemotherapy cycle, she developed acute kidney injury, which was managed with aggressive hydration therapy. However, she subsequently exhibited signs of sepsis and a deteriorating level of consciousness, necessitating admission to the intensive care unit. Despite intensive care, the patient ultimately resulting in multi-organ failure and cardiorespiratory arrest.

## Discussion

Colon cancer is the third most frequently diagnosed cancer globally [1], with adenocarcinoma being the predominant histological type. Cardiac metastasis is an exceptionally rare phenomenon, often diagnosed late or at autopsy. The longest reported interval before cardiac metastasis is 17 years [4]. In our case, cardiac metastasis was identified 19 months after the initial diagnosis.

Clinically, cardiac metastases can be asymptomatic or present with nonspecific symptoms such as dyspnea, palpitations, or signs of heart failure [5]. Our patient presented with progressive exertional dyspnea, a symptom with a broad differential diagnosis, particularly in oncology patients, where dyspnea may result from pulmonary embolism, anemia, heart failure, pleural effusion, pericardial effusion, or treatment-related cardiotoxicity. Notably, pulmonary embolism and intracardiac involvement were diagnosed concurrently, complicating the clinical picture. This case highlights the diagnostic complexity of intracardiac metastases, which can mimic more common cardiovascular conditions, potentially delaying appropriate intervention and management. Imaging modalities, including transthoracic and transesophageal echocardiography, CT, and magnetic resonance imaging (MRI), are essential for diagnosing cardiac metastases. MRI provides superior tissue characterization, but access remains limited in many settings, particularly in resource-constrained environments. [6].

Given the infrequency of cardiac metastasis, there is no established treatment protocol. Most findings stem from autopsy studies, as obtaining histopathological confirmation of cardiac metastasis is challenging. Some reports indicate that chemotherapeutic agents like 5-fluorouracil, oxaliplatin, and irinotecan (FOLFIRINOX) may be beneficial when used in conjunction with vascular endothelial growth factor inhibitors, such as bevacizumab, for treating cardiac tumors [7]. Surgical resection of the tumor, as performed in our patient, has been documented in the literature. Koizumi *et al.* noted that, while surgery is infrequently recommended for metastatic cardiac tumors, it may be effective for obstructive and solitary lesions, offering symptom relief and potentially extending life expectancy [8].

In a comprehensive case review conducted by Sarfraz *et al*., 31 instances of cardiac metastasis originating from colorectal carcinoma were analyzed. Patient ages ranged from 41 to 81 years, with a predominance of male subjects. The interval between the initial diagnosis of the primary colorectal tumor and subsequent cardiac involvement demonstrated considerable variability, extending up to 17 years in some cases. The right atrium emerged as the most frequently affected cardiac chamber, followed by the right ventricle. Clinical manifestations were generally nonspecific and included symptoms such as dyspnea and palpitations, contributing to diagnostic delays. Multimodal imaging—including transthoracic echocardiography, CT, and MRI was instrumental in establishing the diagnosis. Therapeutic strategies varied: 14 patients underwent surgical resection alone, three received chemotherapy alone, four underwent surgery followed by adjuvant chemotherapy, nine received no treatment, and treatment details were unavailable for two patients. Despite intervention, overall prognosis remained poor, with only nine patients reported as alive at the time of follow-up [3]. Kasama *et al*. [9] describe a case of cardiac metastasis occurring 15 years post-colorectal cancer resection, highlighting the potential for late cardiac involvement. Choi *et al*. [2] report a right atrial mass, stressing the importance of considering metastatic disease in cardiac mass diagnosis. Elbatarny *et al*. [10] present a rare case of isolated metastasis to the right ventricle. These reports collectively underscore the diagnostic complexity and suboptimal therapeutic outcomes associated with cardiac metastases from colorectal cancer, highlighting the need for increased clinical vigilance and further research into effective management approaches.

Our patient had a large obstructive lesion in the right ventricle, warranting surgical removal followed by systemic therapy. Unfortunately, her disease progression led to complications, ultimately resulting in her death.

## Conclusion

The rarity of cardiac metastasis from colon adenocarcinoma presents significant challenges in diagnosis and treatment. While prognosis remains poor, advancements in medical imaging and surgical techniques provide avenues for improving patient care and survival outcomes. Future research should focus on standardizing treatment protocols and exploring novel therapeutic approaches for managing cardiac metastases in colorectal cancer patients.

## Data Availability

The data used to support the findings of this study are included within the article.
